# Versorgungssituation von Menschen mit Vitiligo: Eine Pilot- und Machbarkeitsstudie zum leitliniengerechten Management in deutschen Hautarztpraxen

**DOI:** 10.1007/s00105-026-05729-8

**Published:** 2026-07-01

**Authors:** Larissa Mientus, Rachel Sommer, Kerstin Steinbrink, Markus Böhm

**Affiliations:** 1 Klinik für Hautkrankheiten, Universitätsklinikum Münster, Von-Esmarch-Str. 58, 48149 Münster, Deutschland; 2 Institut für Versorgungsforschung in der Dermatologie und bei Pflegeberufen, Universitätsklinikum Hamburg-Eppendorf, Hamburg, Deutschland

**Keywords:** Stigmatisierung, Psychosoziale Komorbidität, Pigmentverlust, Medizinische Versorgung, Leitlinien, Stigmatization, Psychosocial comorbidity, Pigment loss, Health care, Guidelines

## Abstract

**Hintergrund:**

Vitiligo ist eine häufige, chronische Erkrankung, die zu einem Pigmentzellverlust der Haut führt. Obgleich es internationale Konsensusempfehlungen zur Diagnostik und Therapie sowie eine im April 2021 erschienene deutsche AWMF-S1-Leitlinie gibt, existieren keinerlei Daten zur Adhärenz dieser Richtlinien bei niedergelassenen deutschen Dermatologen. Ziel des vorliegenden Pilotprojektes war es, anhand einer Machbarkeitsstudie einen ersten Einblick in die routinemäßige Versorgungssituation von Menschen mit Vitiligo zu gewinnen.

**Methodik:**

In einer querschnittlichen Beobachtungsstudie haben wir 572 niedergelassene Dermatologen aus Deutschland mit einem Fragebogen adressiert und diese zu ihrer Einstellung zur Vitiligo, der leitlinienkonformen Diagnostik sowie den angebotenen erstattungsfähigen Therapieoptionen befragt.

**Ergebnisse:**

Mehrheitlich (53,7 %) wurde Vitiligo als kosmetisches Problem betrachtet. Über die Hälfte (51,1 %) der Hautärzte wendete keine der abgefragten diagnostischen Methoden an, und rund ein Viertel (25,6 %) nutzte keines der abgefragten Therapiemittel. Dermatologen, die Vitiligo als ernste Erkrankung betrachten, verwenden signifikant häufiger Diagnostikverfahren und verordnen signifikant häufiger leitliniengerechte Therapien als jene, die Vitiligo als kosmetisches Problem sehen.

**Schlussfolgerung:**

Unsere querschnittliche Beobachtungs- und Pilotstudie weist auf eine alarmierende Unterversorgung von Patienten mit dem Krankheitsbild einer Vitiligo im niedergelassenen Bereich hin. Weiterführende große und detailliertere Studien in Deutschland sind nötig, um unsere ersten Daten zu untermauern. Zusätzlich braucht es Maßnahmen, um eine leitliniengerechte Behandlung bei Vitiligo zu verbessern.

**Graphic abstract:**

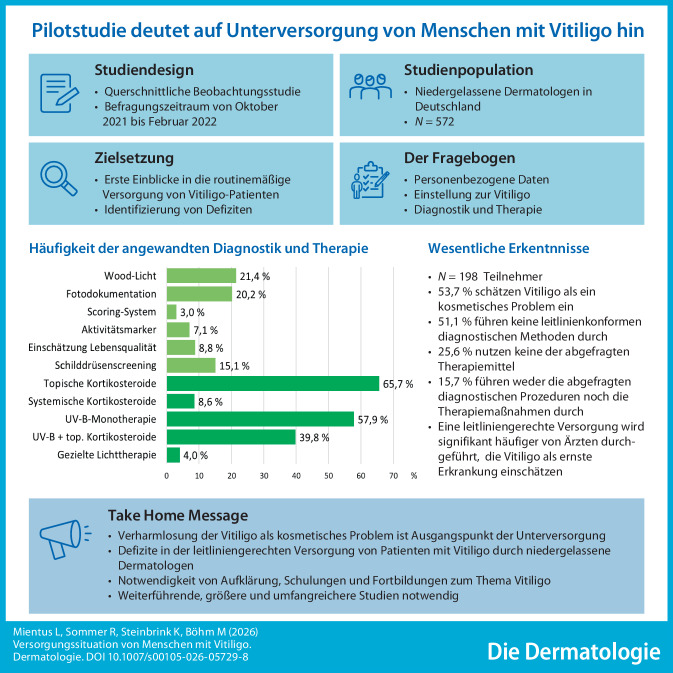

**Zusatzmaterial online:**

Die Online-Version dieses Beitrags (10.1007/s00105-026-05729-8) enthält eine zusätzliche Abbildung. Beitrag und Zusatzmaterial stehen Ihnen im elektronischen Volltextarchiv auf https://www.springermedizin.de/die-dermatologie zur Verfügung. Sie finden das Zusatzmaterial am Beitragsende.

## Abstract (en)

**Background:**

Vitiligo is a common chronic disease leading to pigment cell loss in the skin. Despite international consensus recommendations on diagnosis and treatment and the German AWMF S1-guideline published in April 2021, no data exist on the adherence to these recommendations among German dermatologists in private practice. The aim of this pilot project and feasibility study was to obtain preliminary insight into the medical care situation provided by German dermatology practices to people with vitiligo.

**Methods:**

In a cross-sectional observational study, we addressed 572 dermatologists working in German private practices with a questionnaire on attitude, utilized diagnostic procedures, and prescription of reimbursable therapies.

**Results:**

The majority (53.7%) considered vitiligo a cosmetic problem. Over half (51.1%) of the dermatologists did not use any of the diagnostic methods surveyed, and approximately a quarter (25.6%) did not use any of the therapies surveyed. Dermatologists who regard vitiligo as a serious disease use diagnostic procedures significantly more often and prescribe guideline-based therapies significantly more frequently than those who view vitiligo as a cosmetic issue.

**Conclusion:**

Our cross-sectional observational and pilot study suggests a dramatic undertreatment of patients with vitiligo in private practices in Germany. Larger and more detailed studies are needed to confirm our preliminary data. In addition, measures that improve guideline-compliant therapy are required.

Vitiligo gehört zu den Erkrankungen der Haut mit einem hohen Risiko einer Stigmatisierung. Eine Vielzahl von Studien hat mittlerweile eine klare Beeinträchtigung der Lebensqualität der Betroffenen gezeigt. Umso wichtiger sind daher eine leitliniengerechte Diagnostik und Therapie dieser Erkrankung, um eine Ausdehnung der Vitiligo, Chronifizierung und psychosoziale Komorbidität zu reduzieren. Wie Menschen mit Vitiligo in der Routineversorgung von niedergelassen Dermatologen in Deutschland bezüglich ihrer Erkrankung beraten und behandelt werden, ist kaum erforscht.

Vitiligo ist eine erworbene, chronische Hautkrankheit, die zu einem immunvermittelten Pigmentverlust der Haut führt. Charakteristisch sind depigmentierte, milchig-weiße Makulae. Haare in Form von Leukotrichie sowie Schleimhäute können betroffen sein. Bei der häufigsten Unterform der Vitiligo, der nichtsegmentalen Vitiligo (NSV), sind die weißen Makulae symmetrisch verteilt und bevorzugt an sichtbaren Stellen der Haut wie dem Gesicht, dem Hals oder den Händen lokalisiert. Symptomatisch können eine erhöhte Lichtempfindlichkeit, Juckreiz und Brennen auftreten [5]. Eine Studie zur Häufigkeit der Vitiligo speziell in Deutschland aus dem Jahr 2014 ermittelte eine Prävalenz von 0,17 % und 0,77 % [19].

Die NSV ist kein Lifestyle-Problem, sondern eine chronische Autoimmunerkrankung, die, basierend auf einer genetischen Disposition, zu einer fehlgeleiteten Immunantwort gegen Melanozyten führt [5]. Die NSV kann mit weiteren somatischen Erkrankungen assoziiert sein, zu denen neben Haut‑, Autoimmun- und Bindegewebserkrankungen mitunter auch metabolische Erkrankungen sowie Gehörveränderungen zählen. Es sind hier die Alopecia areata („Odds Ratio“ [OR] = 2,63) und Schilddrüsenerkrankungen (1,61–37 %) wie die Autoimmunthyreoiditis (OR = 10,39) als Beispiele zu erwähnen [17]. Außerdem zeigen Studien eine um etwa 65 % erhöhte Wahrscheinlichkeit für ein metabolisches Syndrom [7] und eine Assoziation zum Diabetes mellitus Typ 2 [6] bei Menschen mit Vitiligo. Dermatologische Erkrankungen wie Psoriasis (OR = 3,22) und atopische Dermatitis (OR = 2,45) sind ebenfalls mit Vitiligo assoziiert [17]. Für ein erhöhtes Risiko von Hautkrebs liegt bei Patienten mit Vitiligo keine übereinstimmende Evidenz vor [18, 20]. Die Kenntnis dieser Begleiterkrankungen ist bedeutsam für die ganzheitliche Betreuung der Menschen mit Vitiligo und erfordert daher ein holistisches Behandlungskonzept.

Bei Vitiligo kann es zudem zu einer starken psychosozialen Beeinträchtigung der Betroffenen kommen. In Abhängigkeit von sowohl der Ethnizität, dem Hauttyp, Alter und Geschlecht der Erkrankten als auch der Ausdehnung und Lokalisation der Läsionen kann es zu einer signifikanten Einschränkung der Lebensqualität sowie zu psychischen Störungen bis hin zu Angsterkrankungen und Depressionen kommen [9]. Insbesondere Patienten mit einem Hauttyp V und VI haben einen höheren Leidensdruck und häufiger eine psychische Komorbidität (73,4 %) [11].

Die Früherkennung mit korrekter Klassifikation der Erkrankung in die prognostisch unterschiedlichen Subtypen, Bestimmung der Lokalisation, Ausdehnung, Aktivität und Ausbreitung im zeitlichen Verlauf (Progression), Einschätzung der psychosozialen Belastung sowie die Einleitung einer leitlinienkonformen und evidenzbasierten Behandlung inklusive supportiver Maßnahmen sind entscheidend, um diese Krankheit optimal zu therapieren. Im April 2021 wurde erstmals die deutsche AWMF-S1-Leitlinie zur rationalen Diagnostik und Therapie der Vitiligo veröffentlicht [3]. Ende 2023 erschienen internationale Konsensusempfehlungen zur Vitiligo, die von der International Vitiligo Task Force erstellt wurden [22, 27]. Außerdem haben wir kürzlich auf die besondere Bedeutung der psychosozialen Komorbidität und auf die verbesserungswürdige Betreuung betroffener Patienten im Rahmen einer holistischen Versorgung hingewiesen [4].

Das Ziel der vorliegenden Pilotstudie ist es, einen ersten Einblick in die leitlinienkonforme Diagnostik und die in deutschen Hautarztpraxen angebotenen erstattungsfähigen In-label-Therapien bei Menschen mit Vitiligo zu gewinnen.

## Material und Methoden

### Studiendesign

Die Studie war ein Pilotprojekt in Form einer querschnittlichen Beobachtungsstudie und sollte als Machbarkeitsstudie Versorgungsdefizite von Menschen mit Vitiligo identifizieren.

### Studienpopulation

Befragt wurden 572 niedergelassene Fachärzte für Haut- und Geschlechtskrankheiten in dermatologischen Praxen in Deutschland. Zur Identifizierung dieser Ärzte wurde der Adressverteiler der Klinik für Hautkrankheiten des Universitätsklinikums Münster benutzt. Es wurden Teilnehmer aus allen Bundesländern eingeschlossen. Der Großteil der Befragten war in Nordrhein-Westfalen (55,2 %, *n* = 316) und Niedersachsen (14,3 %, *n* = 82) niedergelassen. Die Studie wurde von der Ethik-Kommission der Ärztekammer Westfalen-Lippe und der Universität Münster bewilligt (Aktenzeichen 2021-463-f-S).

### Praktische Umsetzung

Ein einseitiger Fragebogen wurde den Teilnehmern nebst Informations- und Einwilligungsschreiben und einem adressierten und frankierten Rückumschlag postalisch zugesandt. Zur Sicherung der Anonymität wurden die Befragten gebeten, nur den ausgefüllten Fragebogen in dem Rückumschlag zurückzusenden. Der Befragungszeitraum erstreckte sich von Oktober 2021 bis Februar 2022.

### Fragebogen

Der Fragebogen beinhaltete Fragen (siehe Zusatzmaterial online Abb. 1), die überwiegend ein dichotomes Antwortmuster (v. a. Ja/Nein-Fragen) hatten, um ein möglichst zügiges Ausfüllen zu ermöglichen. Zusätzlich gab es Fragen mit Mehrfachantworten oder offene Fragen. Am Beginn des Fragebogens wurde nach personenbezogenen Daten und der Einstellung zur Krankheit Vitiligo gefragt. Darauf folgten Fragen zum diagnostischen Vorgehen (Scoring-System, Fotodokumentation, Wood-Licht, Aktivitätsmarker, Blutuntersuchung, Einschätzung der Lebensqualität) bei Vitiligo. Hiernach wurden Fragen zum Therapiemanagement mit dem Fokus auf erstattungsfähige Behandlungen (topische und systemische Kortikosteroide) sowie Phototherapien inklusive derer Kombinationen gestellt. Im Fragebogen haben wir nichterstattungsfähige Therapieoptionen überwiegend nicht adressiert, z. B. topische Calcineurininhibitoren als Monotherapie, Antioxidanzien und chirurgische Interventionen.

### Statistische Auswertung

Zur statistischen Auswertung wurden die Daten aus den Fragebögen in SPSS (IBM SPSS Statistics Version 28.0.0.0, IBM, Armonk, NY, USA) übertragen und mithilfe deskriptiver Statistik (absolute [*n*] und relative Häufigkeiten [%] für kategoriale Variablen; Mittelwerte und Standardabweichungen [M ± SD] für stetige Variablen) beschrieben. Um das Diagnostik- und Therapieverhalten zwischen Dermatologen, die Vitiligo als kosmetisches Problem, und solchen, die sie als ernste Erkrankung betrachten, zu vergleichen, wurden univariate Kovarianzanalysen (ANCOVA) durchgeführt einschließlich Alter und Anzahl Berufsjahre (nur für Kortikosteroide der Klasse III) und Geschlecht (nur für Fotodokumentation) als Kovariaten. Der kritische *p*-Wert (α) = 0,05 wurde als Signifikanzniveau festgelegt. Die Abbildungen wurden mit Microsoft Excel erstellt.

## Ergebnisse

### Rücklaufquote und Handhabung der Fragebögen

Bei einer Rücklaufquote von 34,6 % waren insgesamt 198 Fragebögen auswertbar; 34 Fragebögen wurden unvollständig ausgefüllt. Um den Informationsgewinn zu maximieren, wurden alle beantworten Fragen auch aus den unvollständig ausgefüllten Fragebögen in die Analyse einbezogen.

### Demografie der teilnehmenden Behandler

Es nahmen 102 Männer (52,3 %) und 93 Frauen (47,7 %) teil. Das Durchschnittsalter (SD) der Studienkohorte betrug 55,3 (7,7) Jahre, und die Altersspanne reichte von 32 bis 78 Jahren. Die durchschnittliche Berufserfahrung (SD) lag bei 23,8 (7,6) Jahren mit einem Minimum bei 3 und einem Maximum bei 49 Jahren.

### Einstellung zur Vitiligo

Die Mehrheit (53,7 %) der befragten Dermatologen betrachtete Vitiligo als kosmetisches Problem.

### Von niedergelassenen Dermatologen angewandte Diagnostik

**Abb. 1:**
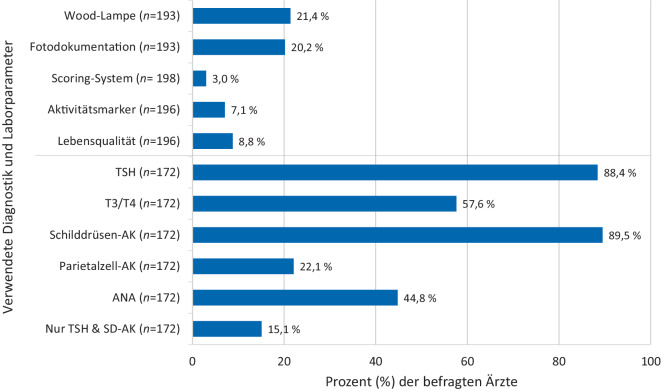
Übersicht der verwendeten Diagnostik und Laborparameter. *TSH* Thyroidea-stimulierendes Hormon, *T3/T4* Thyroxin/Trijodthyronin, *AK* Antikörper, *ANA* antinukleäre Antikörper, *SD* Schilddrüse

Unter den abgefragten diagnostischen Methoden wurde das Wood-Licht nur von einer Minderheit der Behandler (21,4 %) angewandt. Noch weniger Dermatologen (20,2 %) gaben an, eine klinische Fotodokumentation der Vitiligo-Läsionen durchzuführen. Ein validiertes Scoring-System zur Erfassung der Ausdehnung der Vitiligo wurde nur von einem sehr kleinen Bruchteil der Befragten (3,0 %) genutzt. Dabei wurde entweder die befallene Körperoberfläche in Prozent („body surface area“ [BSA]), der „Vitiligo Area Scoring Index“ (VASI) [13] oder der „Vitiligo European Task Force(VETF)-Score“ [24] angegeben. Auf das Vorhandensein von klinischen Parametern einer aktiven Vitiligo prüften ebenfalls wenige Dermatologen (7,1 %). Dabei wurde hauptsächlich auf das Koebner-Phänomen sowie die inflammatorische Vitiligo und Konfetti-Läsionen geprüft. Nur 8,8 % der Behandler schätzten die dermatologische Lebensqualität ein. Die meisten Befragten (86,9 %) gaben an, Blutuntersuchungen bei Menschen mit Vitiligo zu machen. Lediglich ein geringer Anteil davon (15,1 %) screente, wie in der Leitlinie [3] empfohlen, nur auf TSH und Schilddrüsenantikörper (Abb. 1). Kein Behandler nutzte alle, nur ein Behandler 5 und knapp über die Hälfte (51,5 %) nutzten keine der 6 abgefragten, leitlinienkonformen [3] diagnostischen Mittel. Letztere hatten überwiegend (66,3 %) die Meinung, dass die Vitiligo ein kosmetisches Problem sei.

### Von niedergelassenen Dermatologen verwendete Therapien

Bei der Auswahl geeigneter Behandlungsmethoden für Menschen mit Vitiligo zeigte unsere Pilotstudie Differenzen unter den Behandlern. Es gab Patientengruppen, die in einigen Hautarztpraxen keinerlei Therapien erhielten. Dazu zählten mit zunehmender Häufigkeit Patienten mit einem Hauttyp I–II (22,1 %), mit einem Hautbefall über 50 % (31,3 %), Kinder (45,8 %) und Schwangere (84,7 %).

**Abb. 2:**
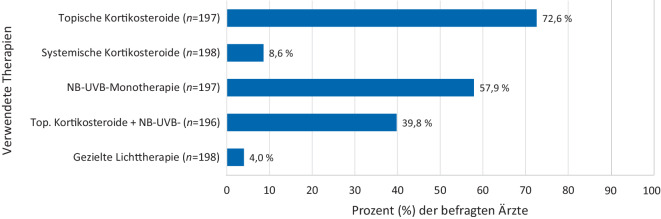
Übersicht der verwendeten Therapien. *NB* Narrow-Band, *UV* ultraviolett

Die Anwendung topischer Kortikosteroide war unterschiedlich; 27,4 % der Dermatologen verschrieben keine topischen Kortikosteroide. Von den übrigen Befragten wurden Kortikosteroide der Klasse II (40,8 %) oder Klasse III (48,5 %) am häufigsten rezeptiert. Mit systemischen Kortikosteroiden wurde selten (8,6 %) behandelt. Eine „Narrow-Band“ (NB, Schmalband)-UVB-Monotherapie wurde von über der Hälfte der befragten Dermatologen genutzt. Knapp 40 % der niedergelassenen Dermatologen gaben an, eine Kombinationstherapie aus NB-UVB und topischen Kortikosteroiden durchzuführen. Eine gezielte Lichttherapie mithilfe eines Excimerlasers wurde sehr selten (4,0 %) angewendet (Abb. 2).

Insgesamt wurde das dermatologische Therapieangebot in den Hautarztpraxen unvollständig ausgeschöpft, da kein Dermatologe alle, nur ein Behandler 4 und rund ein Viertel (25,6 %) keine der angegebenen Behandlungen durchführte. Von den Letzteren vertraten 73,8 % die Meinung, dass die Vitiligo ein kosmetisches Problem sei.

Insgesamt dokumentierten 15,7 % der befragten niedergelassenen Dermatologen, keine der im Fragebogen aufgelisteten diagnostischen Prozeduren und Therapiemaßnahmen bei Menschen mit Vitiligo durchzuführen. Diese Dermatologen waren mehrheitlich (77,4 %) der Meinung, dass die Vitiligo ein kosmetisches Problem sei.

Vergleichende Analysen zeigten signifikante Unterschiede hinsichtlich der Diagnostik und Therapieauswahl zwischen Dermatologen, die Vitiligo als kosmetisches Problem betrachten, und jenen, die Vitiligo als ernste Erkrankung einstufen. Dermatologen, die Vitiligo als kosmetisches Problem erachten, verordnen signifikant häufiger keine Kortikosteroide. Dermatologen, die Vitiligo als ernste Erkrankung betrachten, nutzen eine Fotodokumentation, das Wood-Licht, Scoring-Verfahren zur Ausdehnung der Erkrankung, Blutabnahmen zur Autoimmundiagnostik sowie Methoden zur Erfassung der Lebensqualität signifikant häufiger als diagnostische Mittel als jene, die Vitiligo als kosmetisches Problem erachten. Zusätzlich verordnen diese signifikant häufiger Kortikosteroide der Klasse III, eine NB-UVB-Therapie plus topische Steroide (Tab. 1). In allen weiteren Diagnose- und Therapieverfahren zeigten sich keine signifikanten Unterschiede.

**Tab. 1:** Kovarianzanalyse zu Diagnose- und Therapieverfahren zwischen Dermatologen, die Vitiligo als ernste Erkrankung vs. kosmetisches Problem erachten

	Ernste Erkrankung		Kosmetisches Problem				
	*n*	M ± SD	*n*	M ± SD	F	df	*p*
Fotodokumentation	78	0,27 ± 0,45	90	0,12 ± 0,33	5,98	1 165	0,016
Nutzung Scoringsystem	81	0,07 ± 0,26	94	0,00 ± 0,00	7,43	1 173	0,007
Wood-Licht	32	0,32 ± 0,47	10	0,10 ± 0,30	14,81	1 173	< 0,001
Laborwert TSH	86	0,86 ± 0,35	70	0,70 ± 0,46	6,78	1 173	0,010
Laborwert Schilddrüsen-AK	81	0,86 ± 0,35	94	0,70 ± 0,46	6,78	1 173	0,010
Laborwert ANA	81	0,49 ± 0,50	94	0,28 ± 0,45	9,10	1 173	0,003
Erfassung Lebensqualität	79	0,13 ± 0,34	93	0,03 ± 0,18	5,55	1 170	0,020
Kortikosteroide Klasse III	80	0,49 ± 0,50	92	0,33 ± 0,47	4,72	1 168	0,031
Keine Kortikosteroide	80	0,18 ± 0,38	94	0,31 ± 0,46	4,20	1 172	0,042
NB-UVB	81	0,74 ± 0,44	94	0,48 ± 0,50	13,25	1 173	< 0,001
NB-UVB + top. Steroide	81	0,57 ± 0,50	93	0,29 ± 0,46	14,70	1 172	< 0,001

*M* Mittelwert, *SD* Standardabweichung, *n* Anzahl („number“)

## Diskussion

Die vorliegende Beobachtungsstudie hatte das Ziel – in Form eines Pilotprojektes und als Machbarkeitsstudie – erste Einblicke in die klinische Versorgungssituation von Menschen mit Vitiligo durch niedergelassene deutsche Dermatologen zu gewinnen. Aufgrund des Pilotcharakters dieser Arbeit repräsentieren unsere Ergebnisse die Ansichten von 5,7 % der niedergelassenen Dermatologen (insgesamt 3485 im Jahr 2021) in Deutschland [14].

Es gibt eine Reihe von Gründen für die hier aufgezeigten Versorgungsdefizite. Die Verharmlosung der Vitiligo als kosmetisches Problem, quasi als nicht-behandlungsbedürftiger Zustand ohne Krankheitswert, steht zweifelsohne am Anfang einer unzureichenden Versorgung von Menschen mit Vitiligo durch Behandler. Eine solche Einstellung, die auch in unserer Pilotstudie identifiziert wurde, steht im klaren Widerspruch zur autoimmunen Genese dieser chronischen Krankheit und selbstverständlich auch zur Definition der Weltgesundheitsorganisation von „Gesundheit als Zustand des vollständigen körperlichen, geistigen und sozialen Wohlergehens“ [16]. Besonders im Hinblick auf die mittlerweile belegte psychosoziale Komorbidität [4] ist Vitiligo nicht selten als schwere und grundsätzlich behandlungsbedürftige Erkrankung einzustufen. Eine nicht-leitlinienkonforme Behandlung, die sich durch eine unzureichende Diagnostik aufgrund des Verzichts auf die Nutzung Diagnose-erleichternder Instrumente wie Wood-Licht, Einteilung der Vitiligo in prognostisch relevante Subtypen, Bestimmung von Ausdehnung und Aktivität, Einschätzung des Leidensdrucks mit validierten PROMs („patient-reported outcome measures“) und durch ein limitiertes therapeutisches Angebot auszeichnet, wird daher zwangsläufig zu einem negativen Verlauf der Patientenkarriere führen, was die mittlerweile bekannten psychosozialen Auswirkungen von Vitiligo noch weiter aggraviert [4]. Eine rezente internationale Studie (VALIANT), in der 3541 Patienten sowie 1203 Health Care Professionals (HCPs) aus unterschiedlichen Ländern befragt wurden, ergab, dass 56,7 % der von Vitiligo Betroffenen mitgeteilt wurde, ihre Erkrankung sei nicht zu behandeln [12]. Die durchschnittliche Zeit von den ersten Symptomen bis zur Diagnosestellung, inklusive anfänglicher Fehldiagnosen, betrug im Mittel 2,4 Jahre [12]. Diese Beobachtungen unterstreichen die Ergebnisse unserer Pilotstudie, die nicht nur die optimierungsbedürftige Einstellung niedergelassener Dermatologen aufdeckt, sondern auch eine damit einhergehende unvollständige leitlinienkonforme Diagnostik und Therapie aufzeigt. Wenn neben der Überprüfung auf klinische Aktivitätsmarker kein objektives Scoring-System zur Bestimmung der Vitiligo-Ausdehnung zusätzlich benutzt wird, ist eine objektive Einordnung der Vitiligo in eine aktive oder stabile Erkrankung schwierig, was zwangsläufig die Therapieentscheidung beeinflusst. Unsere Daten zeigen, dass ein Großteil der diagnostischen Verfahren signifikant häufiger durch niedergelassene Dermatologen eingesetzt wird, die Vitiligo als ernste Erkrankung betrachten. In Übereinstimmung konnten wir feststellen, dass niedergelassene Dermatologen, die Vitiligo als ernste Erkrankung einschätzen, zur Verfügung stehende Therapien signifikant häufiger verordnen als jene, die Vitiligo als kosmetisches Problem betrachten.

Mittlerweile gibt es neben der diagnostischen Basisuntersuchung zur Bestimmung der betroffenen BSA bei der Vitiligo benutzerfreundliche App-basierte (https://www.vitiligo-calculator.com/) Scoring-Systeme wie den Vitiligo Extent Score [25] und Self-Assessment-Vitiligo Extent Score [26]. Wir haben kürzlich anhand einer Checkliste auf die Wichtigkeit der exakten Bestimmung der Vitiligo-Ausdehnung, insbesondere im Rahmen des neu zugelassenen topischen Januskinaseinhibitors Ruxolitinib [21], hingewiesen [2].

Im Gegensatz zur Psoriasis vulgaris und atopischen Dermatitis, die bei Verschreibung kostenintensiver Biologika die Einschätzung der Lebensqualität mit dem Dermatology Life Quality Index erfordern, ist die Bestimmung der psychosozialen Belastung bei Menschen mit Vitiligo, wie auch unsere Pilotstudie zeigt, noch nicht routinemäßig etabliert. Im Rahmen eines Positionspapieres zur Einschätzung der psychosozialen Belastung und etwaigen Komorbidität (Angsterkrankung, Depression) haben wir kürzlich auf einfache Screeningmethoden bei Menschen mit Vitiligo diesbezüglich hingewiesen [4].

Allein durch lückenhafte Diagnostik wird sowohl für den Behandler als auch den Betroffenen die Aufstellung eines gemeinsam definierten Therapieziels, d. h. Stopp der Progression, Repigmentierung oder Stabilisierung der Erkrankung, und der sich daraus ergebenden besten Therapie gemäß einer partizipativen Therapieentscheidung („Shared Decision-Making“) [23], höchstwahrscheinlich undurchsichtig. Dies könnte zu den in dieser Pilotstudie identifiziertem Behandlungsdefiziten von Menschen mit Vitiligo möglicherweise beitragen, die auch in Studien anderer Länder nachgewiesen wurden [1, 8, 10].

Weitere Gründe, die zu der hier aufgezeigten Unterversorgung führen, könnten eine unzureichende Schulung niedergelassener Dermatologen bezüglich der rationellen Diagnostik und des evidenzbasierten Therapiemanagements der Vitiligo, ungenügende Verfügbarkeit von Therapiegeräten (v. a. UV-Bestrahlungsgeräte), finanzielle Aspekte der Vergütungssystematik (z. B. die in der Regel nicht kassenärztlich erstattungsfähige gezielte Bestrahlung mit dem Excimer-Laser oder der -Lampe) oder eine mangelnde Compliance der betroffenen Patienten sein.

Die anfangs beschriebene Verharmlosung der Vitiligo als kosmetisches Problem ist in Anbetracht der hier identifizierten Versorgungsdefizite a priori ein fundamentaler Faktor, der über unzureichende Krankheits-„Awareness“ direkt zur Unterversorgung und Nichtadhärenz bestehender Leitlinien führen dürfte. Dies zeigt daher die Notwendigkeit einer umfassenden „Awareness“-Arbeit, damit Vitiligo als Erkrankung ernst genommen wird und Patienten im Sinne einer patientenorientierten Versorgung die Therapie erhalten, die sie benötigen.

Unsere systematischen Daten zur Versorgungssituation von Menschen mit Vitiligo in deutschen Hautarztpraxen weisen erstmalig auf relevante Defizite in der Diagnostik und Therapie dieser Krankheit hin. Trotz der nachfolgend aufgeführten Limitationen hat unsere Pilotstudie eine hohe berufs- und versorgungspolitische Bedeutung und stellt einen Ausganspunkt für eine bundesweite, flächige Versorgungsstudie dar. Die im Rahmen des Hautnetz-Deutschlands am 07.07.2026 einberufene erste Nationale Versorgungskonferenz Vitiligo [15] wird eine Plattform bieten, die Versorgungssituation von Personen mit Vitiligo systematisch und flächendeckend in Deutschland anzugehen.

### Limitationen

Folgende Änderungen wären nötig, um die Bedeutsamkeit der Studienergebnisse zu erhöhen:

- Vergrößerung der Stichprobe,
- flächendeckende Befragung von Behandlern in Deutschland ohne geografische Überrepräsentation einzelner Regionen,
- Erweiterung der Befragung von Dermatologen in Kliniken,
- Erweiterung der Befragung um die sehr wichtige Frage, ob die seit April 2023 offiziell für die NSV zugelassene Therapie mit topischem Ruxolitinib von niedergelassen Dermatologen verschrieben wird,
- Erweiterung der Befragung auf eine Reihe von nichterstattungsfähigen Therapien wie topische Calcineurininhibitoren (z. B. als Monotherapie), topische und systemische Antioxidantien und chirurgische Verfahren sowie supportive Maßnahmen (z. B. SDM, Vermittlung psychotherapeutischer Angebote, Anbindung an Patientenorganisationen, Camouflage),
- Erweiterung und Bestätigung unserer präliminären Daten durch Analyse von Schadensdatenbanken („claims data analysis“).

## Fazit für die Praxis

- Unter Berücksichtigung einer möglichen geografischen Verzerrung deuten unsere Ergebnisse auf eine Unterversorgung der Menschen mit Vitiligo im deutschsprachigen Raum hin, was sowohl die Diagnostik als auch die Therapie dieser oft stigmatisierenden Krankheit betrifft.
- Es besteht die Notwendigkeit eines erhöhten Bewusstseins für Vitiligo als relevante Hauterkrankung.
- Es besteht dringender Bedarf einer optimierten klinischen Ausbildung niedergelassener Dermatologen durch regelmäßige Schulungen und Fortbildungen zum Thema Vitiligo.

## Supplementary Information

Online-Abb. 1 Fragebogen zur Diagnostik und Therapie der Vitiligo

## Data Availability

Die in dieser Studie erhobenen Datensätze können auf begründete Anfrage beim Korrespondenzautor angefordert werden.

## References

[1] Alghamdi KM (2009) A survey of vitiligo management among dermatologists in Saudi Arabia. J Eur Acad Dermatol Venereol 23:1282–1288 19522714 10.1111/j.1468-3083.2009.03310.x

[2] Augustin M, Böhm M, Berneburg M et al (2024) Vitiligo: Krankheitslast erfordert medizinische Versorgung. Berufspolitik 72:20–25

[3] Böhm M, Schunter JA, Fritz K et al (2022) S1-Leitlinie: Diagnostik und Therapie der Vitiligo. J Dtsch Dermatol Ges 20:365–379 35304960 10.1111/ddg.14713_g

[4] Böhm M, Sommer R, Gieler U et al (2024) Krankheit Vitiligo. Ein Positionspapier zur Stigmatisierung, Einschränkung der Lebensqualität und psychosozialen Komorbidität. J Dtsch Dermatol Ges 22:1327–133539167551

[5] Böhm M, Tanew A (2025) Vitiligo. J Dtsch Dermatol Ges 23:968–987 40788636 10.1111/ddg.15706 PMC12338405

[6] Chang HC, Lin MH, Huang YC et al (2019) The association between vitiligo and diabetes mellitus: A systematic review and meta-analysis. J Am Acad Dermatol 81:1442–1445 31228523 10.1016/j.jaad.2019.06.022

[7] Chuang KW, Chang HC (2022) Association between vitiligo and metabolic syndrome: A systematic review and meta-analysis. J Dtsch Dermatol Ges 20:218–221 34990080 10.1111/ddg.14652

[8] Eleftheriadou V, Delattre C, Chetty-Mhlanga S et al (2024) Burden of disease and treatment patterns in patients with vitiligo: findings from a national longitudinal retrospective study in the UK. Br J Dermatol 191:216–224 38534198 10.1093/bjd/ljae133

[9] Ezzedine K, Eleftheriadou V, Jones H et al (2021) Psychosocial Effects of Vitiligo: A Systematic Literature Review. Am J Clin Dermatol 22:757–774 34554406 10.1007/s40257-021-00631-6 PMC8566637

[10] Ezzedine K, Seneschal J, Da Silva A et al (2023) Vitiligo patient population an disease burden in France: VIOLIN study results from CONSTANCES cohort. J Eur Acad Dermatol Venereol 37:2249–2258 37605309 10.1111/jdv.19447

[11] Grimes P, Hamzavi IH, Bibeau K et al (2025) Mental Health and Psychosocial Burden Among Patients with Skin of Color Living with Vitiligo: Findings from the Global VALIANT Study. Dermatol Ther (Heidelb) 15:1931–1939 40325321 10.1007/s13555-025-01412-3 PMC12126399

[12] Hamzavi IH, Bibeau K, Grimes P et al (2023) Exploring the natural and treatment history of vitiligo: perceptions of patients and healthcare professionals from the global VALIANT study. Br J Dermatol 189:569–577 37493275 10.1093/bjd/ljad245

[13] Hamzavi I, Jain H, McLean D et al (2004) Parametric modeling of narrowband UV‑B phototherapy for vitiligo using a novel quantitative tool: The Vitiligo Area Scoring Index. Arch Dermatol 140:677–683 15210457 10.1001/archderm.140.6.677

[14] https://www.bundesaerztekammer.de/fileadmin/user_upload/BAEK/Ueber_uns/Statistik/2021/2021_Statistik.pdf. Zugegriffen: 9. März 2025

[15] https://www.hautnetz-deutschland.de/events/1-nationale-versorgungskonferenz-vitiligo-2026-hybrid/ Zugegriffen 16. März 2026

[16] https://www.who.int/about/governance/constitution Zugegriffen: 12. Januar 2025

[17] Lee JH, Ju HJ, Seo JM et al (2023) Comorbidities in Patients with Vitiligo: A Systematic Review and Meta-Analysis. J Invest Dermatol 143:777–789.e6 36574529 10.1016/j.jid.2022.10.021

[18] Long VS, Law YXT, Yew YW, Choi CE (2022) Risk of internal malignancies, cutaneous malignant melanoma and non-melanoma skin cancer among patients with vitiligo: a systematic review and meta-analysis. J Eur Acad Dermatol Venereol 36:e829–e831 35686630 10.1111/jdv.18310

[19] Mohr N, Petersen J, Kirsten N, Augustin M (2021) Epidemiology of vitiligo - a dual population-based approach. Clin Epidemiol 13:373–382 34079380 10.2147/CLEP.S304155 PMC8165096

[20] Rooker A, Ouwerkerk W, Bekkenk MW et al (2024) The risk of keratinocyte cancer in Vitiligo and the potential mechanisms involved. J Invest Dermatol 144:234–242 37791932 10.1016/j.jid.2023.08.012

[21] Rosmarin D, Passeron T, Pandya AG et al (2022) Two phase 3, randomized, controlled trials of ruxolitinib cream for vitiligo. N Engl J Med 387:1445–1455 36260792 10.1056/NEJMoa2118828

[22] Seneschal P, Speeckaert R, Taïeb A et al (2023) Worldwide expert recommendations for the diagnosis and management of vitiligo: Position statement from the international Vitiligo Task Force-Part 2: Specific treatment recommendations. J Eur Acad Dermatol Venereol 37:2185–2195 37715487 10.1111/jdv.19450

[23] Shourick J, Ahmed M, Seneschal J et al (2021) Development of a shared decision-making tool in vitiligo: an international study. Br J Dermatol 185:787–796 33830502 10.1111/bjd.20137

[24] Taïeb A, Picardo M et al (2007) The definition and assessment of vitiligo: a consensus report of the Vitiligo European Task Force. Pigment Cell Res 20:27–35 17250545 10.1111/j.1600-0749.2006.00355.x

[25] van Geel N, Lommerts J, Bekkenk M et al (2016) Development and Validation of the Vitiligo Extent Score (VES): an International Collaborative Initiative. J Invest Dermatol 136:978–984 26827762 10.1016/j.jid.2015.12.040

[26] van Geel N, Lommerts JE, Bekkenk MW et al (2017) Development and validation of a patient-reported outcome measure in vitiligo: The Self Assessment Vitiligo Extent Score (SA-VES). J Am Acad Dermatol 76:464–471 27887798 10.1016/j.jaad.2016.09.034

[27] van Geel N, Speeckaert R, Taïeb A et al (2023) Worldwide expert recommendations for the diagnosis and management of vitiligo: Position statement from the International Vitiligo Task Force Part 1: towards a new management algorithm. J Eur Acad Dermatol Venereol 37:2173–2184 37746876 10.1111/jdv.19451

