# Supplementary material for: Medical care situation of people with vitiligo: A pilot and feasibility study on guideline-oriented management in German dermatology practices
Source: Dermatologie (Heidelb). 2026 Jul 1;77(8):524–30. [Article in German] doi: 10.1007/s00105-026-05729-8 (PMC13433705; doi:10.1007/s00105-026-05729-8)
Supplement: Supplementary file 1 [file 105_2026_5729_MOESM1_ESM.pdf]

Online-Abbildung 1

Fragebogen zur Diagnostik und Therapie der Vitiligo

1. Bitte geben Sie Ihr Alter an: _____ Jahre

2. Bitte geben Sie Ihr Geschlecht an: ☐ männlich ☐ weiblich ☐ keine Angabe

3. Seit wie vielen Jahren sind Sie als Hautarzt/Hautärztin tätig? Seit _____ Jahren

4. Betrachten Sie die Vitiligo eher als ein kosmetisches Problem oder eine ernste Erkrankung?
   ☐ Kosmetisches Problem ☐ Ernste Erkrankung

5. Benutzen Sie ein Scoring-System zur Bestimmung des Ausbreitungsgrades der Vitiligo?
   ☐ Ja ☐ Nein
   Wenn Ja, welche/s? ____

6. Führen Sie zur Erfassung des Krankheitsverlaufs eine Fotodokumentation durch?
   ☐ Ja ☐ Nein

7. Nutzen Sie Wood-Licht bei der Diagnosestellung?
   ☐ Ja ☐ Nein

8. Prüfen Sie auf Aktivitätsmarker der Vitiligo?
   ☐ Ja ☐ Nein
   Wenn Ja, auf welche/n? ____

9. Welche Laborwerte überprüfen Sie bei der Vitiligo?
   ☐ TSH ☐ T3/T4 ☐ Schilddrüsen-AK ☐ Parietalzell-AK ☐ ANA ☐ ____________

10. Schätzen Sie die Lebensqualität der Vitiligo-Patienten anhand einer Skala ein?
    ☐ Ja ☐ Nein

11. Wann behandeln Sie einen Vitiligo-Patienten NICHT?
    ☐ Hauttyp I-II ☐ Kinder ☐ __________
    ☐ Ausbreitung > 50% ☐ bei Schwangerschaft

12. Welches topisches Kortikosteroid rezeptieren Sie am häufigsten?
    ☐ Klasse 1 ☐ Klasse 2 ☐ Klasse 3 ☐ Klasse 4 ☐ Keine

13. Nutzen Sie systemische Steroide zur Behandlung der Vitiligo?
    ☐ Ja ☐ Nein

14. Behandeln Sie die Vitiligo mit UVB-311nm?
    ☐ Ja ☐ Nein

15. Führen Sie eine Kombinationstherapie von UVB und topischen Steroiden durch?
    ☐ Ja ☐ Nein

16. Wenden Sie eine gezielte Lichttherapie mittels 308-nm-Excimer-Laser bzw. -Lampe an?
    ☐ Ja ☐ Nein
